# Sensitization to 19 allergen sources in 2,124 children in Kashi Prefecture, China: a single-center cross-sectional retrospective study

**DOI:** 10.3389/falgy.2026.1795685

**Published:** 2026-04-29

**Authors:** Riziwanguli Maitusong, Asimuguli Wubuli, Ayiguli Saimaier, Lifang Li, Gumina Kamili, Muyesaier Maimaiti, Rexiati Dawuti

**Affiliations:** 1 The Second Department of Pediatric Internal Medicine, The First People’s Hospital of Kashi, Kashi, Xinjiang, China; 2 Department of Medical Laboratory, The First People’s Hospital of Kashi, Kashi, Xinjiang, China; 3 Pediatric Center, The First People’s Hospital of Kashi, Kashi, Xinjiang, China

**Keywords:** allergic diseases, child, immunoglobulin E, Kashi Prefecture, sensitization

## Abstract

**Objectives:**

To investigate the sensitization to common allergen sources among children in Kashi Prefecture and offer a scientific foundation for preventing allergic diseases in this demographic.

**Methods:**

The study retrospectively collected the detection results of serum allergen-specific IgE antibodies from 2,124 children at the First People's Hospital of Kashi Prefecture from January 2022 to December 2024.

**Results:**

The overall sensitization rate detected by sIgE was 42.70%. Within the sensitization cases, inhalant allergens had a sensitization rate of 29.52%. The most frequent allergen sources included tree combinations (330 cases, 15.54%), cat fur (243, 11.44%), and *Artemisia argyi* (195, 9.18%). The sensitization rate for food allergens was 24.01%, with egg whites (229, 10.78%), milk (130, 6.12%), and peanuts (99, 4.66%) being the most frequent sources. Sensitization to tree combinations, *Ambrosia artemisiifolia*, cat fur, and peanuts was significantly higher in boys compared to girls. Tree combinations, *Ambrosia artemisiifolia*, *Artemisia argyi*, house dust, cat fur, mold combinations, and *Humulus scandens* exhibited an increasing trend in sensitization rate with age, whereas egg whites, milk, and beef exhibited decreasing trends with age. The sensitization rates for tree combinations, house dust, and mutton displayed significant seasonal variations.

**Conclusions:**

Sensitization to common allergens was prevalent among children in Kashi, although only a small number exhibited allergic symptoms. The sensitization rates for common allergens varied significantly based on sex, age and season. These results suggest that sIgE detection of specific allergens can aid in identifying triggering factors, which is crucial for preventing allergic diseases.

## Introduction

The prevalence of allergic diseases is a major global health challenge, placing a considerable burden on both developed and emerging economies (1, 2). Unfortunately, the prevalence of these diseases continues to rise (2–4), with a particularly high incidence in childhood (4, 5). In fact, allergies and related diseases affect approximately 30% of the population and nearly 80% of families (1). Common clinical allergic diseases include allergic rhinitis, bronchial asthma, atopic dermatitis, and allergic gastroenteritis (1, 6, 7). These diseases are primarily caused by genetic predisposition and exposure to environmental allergens and irritants (2).

Allergic diseases are primarily caused by type I allergic reactions mediated by immunoglobulin E (IgE) (2, 8, 9). Two main methods for identifying allergen sensitization and detecting specific IgE are the skin prick test (SPT) and serum-specific IgE (sIgE) assay. The SPT involves injecting small amounts of allergen into the body to observe the skin reaction. It is a rapid and sensitive test, but it requires the cooperation of the child and carries a risk for highly sensitized individuals. On the other hand, the sIgE assay is stable, unaffected by medications, and has a low risk profile. It helps to identify the cause of diseases and the level of sensitization (9). Due to differences in geography, ethnicity, living habits, and dietary patterns, the pathogenicity of allergens varies in different regions. Therefore, it is crucial to analyze the epidemiological investigation of allergic diseases in different regions.

Kashi Prefecture is situated in the southwest of the Xinjiang Uygur Autonomous Region, China. It is bordered by the Taklamakan Desert to the east, the Tianshan Mountains to the north, the Pamirs Plateau to the west, and the Karakoram Mountains to the south. The prefecture covers an area of 162,000 km^2^ and has a population of over 4,488,200 people residing in 12 cities and counties (10). The climate of Kashi Prefecture is desert continental, with obvious seasonal changes (11). This leads to obvious changes in temperature, rainfall and vegetation in different seasons (11) and may have different effects on allergic diseases of children. Therefore, studying allergen sensitization in children in Kashi Prefecture offers valuable information for prevention of allergic diseases in the region. In this study, we collected clinical data from children at the First People's Hospital of Kashi Prefecture between January 2022 and December 2024, and analyzed the sensitization of 19 allergens among them. Our study serves as a reference for the prevention of children's allergic diseases in Kashi Prefecture.

## Material and methods

### Ethics statement

The study was approved by the Ethics Committee of the First People's Hospital of Kashi Prefecture [(2022) Kuaishenyan No. 32], and conducted in accordance with the guidelines outlined in the Declaration of Helsinki. Informed consent obtained from the study participants (minors) parents/guardians prior to study commencement.

### Research object and inclusion and exclusion criteria

A cross-sectional retrospective study was conducted to analyze the clinical data of children who had completed sIgE detection in the outpatient and emergency departments, as well as in-patients at the First People's Hospital of Kashi Prefecture from January 2022 to December 2024. The inclusion criteria were as follows: (1) Children aged 0–14 years old; (2) Children with sIgE detection data. The exclusion criteria were: (1) Incomplete case information; (2) Children with dwarfism, leukemia, fractures, brucellosis, intestinal obstruction, herpes zoster, seizures of unknown cause, and diseases that cannot be accurately diagnosed. (3) Failure to sign the informed consent form or refusal from the guardian to participate in the study.

### Disease diagnosis

Allergic diseases, including allergic rhinitis, asthmatic pneumonia, asthmatic bronchopneumonia, asthmatic bronchitis, acute asthmatic bronchitis, anaphylaxis, allergic cough, chronic nasosinusitis, chronic rhinitis, chronic cough, allergic dermatitis, rash, eczema, universal eczema, asthma, cough-variant asthma, bronchial asthma, severe asthma, urticaria, acute urticaria, chronic urticaria, papular urticaria, allergic pharyngitis, prurigo, acute prurigo, nodular prurigo, Henoch-Schönlein purpura, mixed allergic purpura, and Henoch-Schönlein purpura nephritis, were diagnosed following the consensus on diagnosis and management of allergic diseases in children (12–19).

Respiratory infectious diseases (RIDs, including pertussis, pneumonia, *Mycoplasma pneumoniae* pneumonia (MP), respiratory tract infection, upper respiratory tract infection (URTI), acute upper respiratory tract infection (AURTI), bronchopneumonia, bronchiectasis with infection, bronchitis, acute bronchitis, and severe pneumonia, as well as viral skin diseases, including viral rash and viral eczema, were diagnosed in accordance with World Health Organization guidelines (20–23).

Additionally, healthy children who underwent health check-up during the same period were included as a healthy baseline.

### Clinical data collection

The clinical data, including basic information such as the children's sex, age, and date of examination, as well as the results of inhalation and food allergen-sIgE detection, were collected for subsequent analysis.

### Serum allergen sIgE test

The children's venous blood specimens were collected by nurses, and the sIgE antibodies for inhalant and food allergens were detected using a sIgE test kit (immunoblotting method) (EUROIMMUN, Lübeck, Germany). The sources of inhalant allergens detected included ten categories: tree combinations (*Salix* sp., *Polulus* sp., and *Ulmus pumila*), *Ambrosia artemisiifolia*, *Artemisia argyi*, combinations of indoor *Dermatophagoides* (CID), house dust, cat fur, dog epithelium, *Periplaneta americana*, mold combinations (*Penicillium notatum*, *Cladosporium cladosporioides*, *Aspergillus fumigatus*, and *Alternaria alternata*), and *Humulus scandens*. The sources of food allergens detected included nine categories: egg whites, milk, peanuts (*Arachis hypogaea*), soybeans (*Giycine max*), beef, seafood combinations (*Acipenser* sp., Palinuridae, and Pectinidae), shrimps, crabs, and mutton. The allergen sIgE antibody concentrations were categorized into six levels, with L0 being negative and L1 to L6 being sensitization. The higher level indicated the stronger degree of sensitization.

### Data analysis

Quantitative data are presented as mean ± standard deviation, whereas qualitative data are presented as number (positive rate). To investigate differences in the distribution of sensitization cases with specific allergen among all children, the chi-squared test was performed for qualitative data in these groups. The 95% confidence interval (CI) of the sensitization rate was calculated using the Compositional package version 7.6 of R. A *P-*value < 0.05 was considered statistically significant.

## Results

### Demographic characteristics

**Figure 1:**
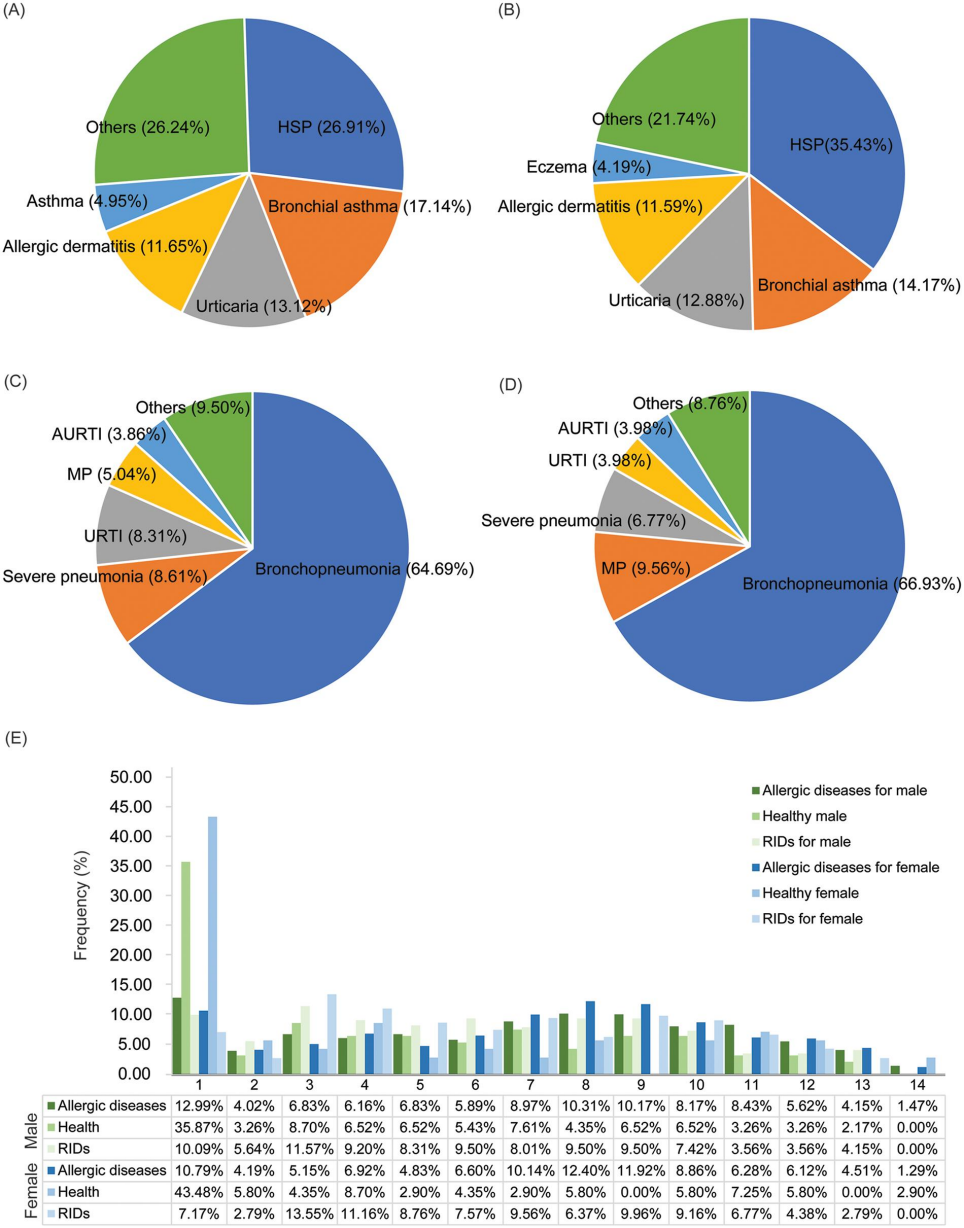
The top five diseases diagnosed in the children included in the study and their proportion and distribution of children in different ages. **(A)** Allergic diseases for male; **(B)** Allergic diseases for female; **(C)** Respiratory infectious diseases for male; **(D)** Respiratory infectious diseases for female; **(E)** Distribution of children in different ages. HSP, Henoch-Schönlein purpura; MP, *Mycoplasma pneumoniae* pneumonia; URTI, Upper respiratory tract infection; AURTI, acute upper respiratory tract infection.

Out of the 2,124 children enrolled, 1,181 (55.60%) were male and 943 (44.40%) were female. Among the boys, 747 were diagnosed with allergic diseases, 337 were diagnosed with respiratory infectious diseases, and 5 were diagnosed with viral rash. Additionally, 92 healthy boys were included. Among the girls, 621 were diagnosed with allergic diseases, 251 were diagnosed with respiratory infectious diseases, one was diagnosed with viral rash, and one was diagnosed with viral eczema. Additionally, 69 healthy girls were included (Supplementary Table S1). The top five allergic diseases diagnosed in the male and female children were Henoch-Schönlein purpura (*n* = 201, 26.91% of total cases of allergic diseases for male, and *n* = 220, 35.43% for female), bronchial asthma (*n* = 128, 17.14% for male, and *n* = 88, 14.17% for female), urticaria (*n* = 98, 13.12% for male, and *n* = 80, 12.88% for female), allergic dermatitis (*n* = 87, 11.65% for male, and *n* = 72, 11.59% for female), and asthma (*n* = 37, 4.95%) for male and eczema (*n* = 26, 4.19%) for female (Figures 1A,B, and Supplementary Table S1). The top five respiratory infectious diseases diagnosed in the male and female children were bronchopneumonia (*n* = 218, 64.69% for male, and *n* = 168, 66.93% for female), severe pneumonia (*n* = 29, 8.61% for male, and *n* = 17, for 6.77% female), URTI (*n* = 28, 8.31% for male, and *n* = 10, 3.98% for female), MP (*n* = 17, 5.04% for male, and *n* = 24, 9.56% for female), and AURTI (*n* = 13, 3.86% for male, and *n* = 10, 3.98% for female). The distribution of allergic (*χ*^2^ test, *χ*^2^ = 35.12, *P* = 0.166) and respiratory infectious (*χ*^2^ test, *χ*^2^ = 12.68, *P* = 0.242) diseases between sexes was not significantly different (Figures 1A–D).

The children were divided into three age groups: 535 (25.27%) in the toddler group (0 to ≤3 years old), 443 (20.93%) in the preschool group (4 to ≤6 years old), and 1,139 (53.80%) in the school-age group (7 to ≤14 years old; Supplementary Table S2). There was no significant difference in the distribution of all cases (*χ*^2^ test, *χ*^2^ = 3.598, *P* = 0.166), allergic cases (*χ*^2^ test, *χ*^2^ = 3.163, *P* = 0.206), healthy cases (*χ*^2^ test, *χ*^2^ = 0.539, *P* = 0.764), or respiratory infectious cases (*χ*^2^ test, *χ*^2^ = 1.153, *P* = 0.562) among age groups between sexes (Figure 1E). The children were also divided into four groups based on the season of consultation: 665 cases (31.31%) in the spring group (March to May), 494 cases (23.26%) in the summer group (June to August), 560 cases (26.37%) in the autumn group (September to November), and 405 cases (19.07%) in the winter group (December to February) (Supplementary Table S3). There was no significant difference in the distribution of all cases (*χ*^2^ test, *χ*^2^ = 1.432, *P* = 0.698), allergic cases (*χ*^2^ test, *χ*^2^ = 0.382, *P* = 0.944), healthy cases (*χ*^2^ test, *χ*^2^ = 0.320, *P* = 0.956), or respiratory infectious cases (*χ*^2^ test, *χ*^2^ = 2.085, *P* = 0.555) among seasons between boys and girls (Supplementary Table S3).

### Compositions of allergen sources detected through sIgE

**Figure 2:**
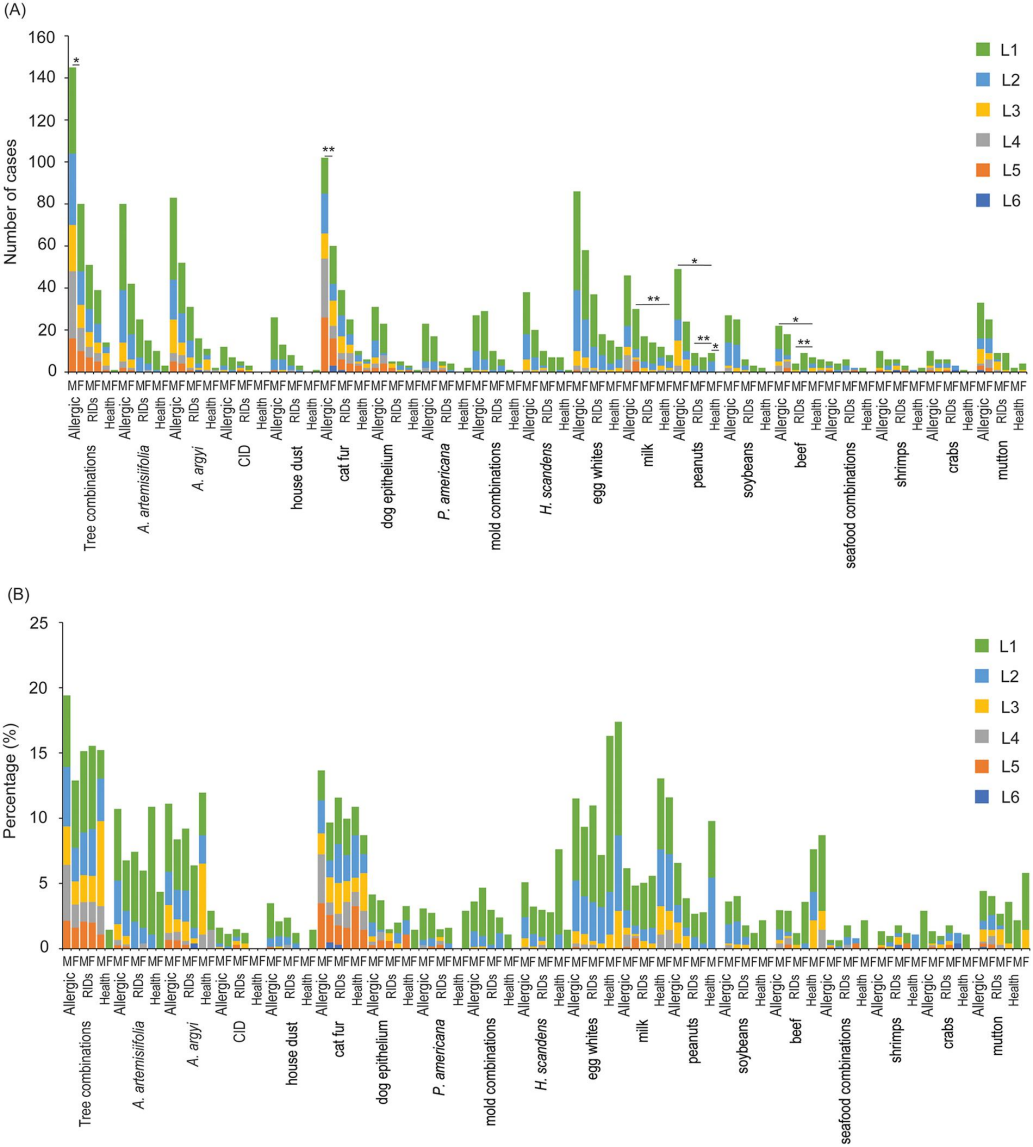
Compositions of allergen levels for each allergen sources. **(A)** Sensitization cases of allergen levels for each allergen source. **(B)** Sensitization percentage of allergen levels for each allergen source. * *P* < 0.05; ** *P* < 0.01.

The overall sensitization rate was 42.70%. The sensitization rates for allergic, respiratory infectious, and healthy cases were 43.71%, 39.80%, and 43.48%, respectively. The sensitization rate for inhalant allergens was 29.52%, with the highest rates found in tree combinations [330 (15.54%)], cat fur [243 (11.44%)], and *Artemisia argyi* [195 (9.18%)]. For allergic cases, the overall sensitization rate was 31.58%, with the highest rates found in tree combinations [225 (16.45%)], cat fur [162 (11.84%)], and *Artemisia argyi* [135 (9.87%)]. For respiratory infectious cases, the overall sensitization rate was 27.23%, with the highest rates found in tree combinations [90 (15.13%)], cat fur [65 (10.92%)], and *Artemisia argyi* [47 (7.90%)]. For healthy cases, the overall sensitization rate was 20.50%, with the highest rates found in cat fur [16 (9.94%)], tree combinations [15 (9.32%)], *Artemisia argyi* [13 (8.07%)], and *Ambrosia artemisiifolia* [13 (8.07%)] (Figure 2). The sensitization rate for food allergens was 24.01%, with the highest rates found in egg whites [229 (10.78%)], milk [130 (6.12%)], and peanuts [99 (4.66%)]. For allergic cases, the overall sensitization rate was 24.34%, with the highest rates found in egg whites [144 (10.53%)], milk [76 (5.56%)], and peanuts [73 (5.34%)]. For respiratory infectious cases, the overall sensitization rate was 21.28%, with the highest rates found in egg whites [58 (9.75%)]. milk [34 (5.71%)], and mutton [19 (3.19%)]. For healthy cases, the overall sensitization rate was 31.68%, with the highest rates found in egg whites [27 (16.77%)]. milk [20 (12.42%)], and beef [13 (8.07%)] (Figure 2). Among the sensitization patients, the L1 level was the most prevalent (Figure 2). Additionally, the sensitization level compositions of tree combinations (*χ*^2^ test, *χ*^2^ = 14.671, *P* = 0.012) and cat fur (*χ*^2^ test, *χ*^2^ = 20.498, *P* = 0.002) were significant difference between allergic males and females (Figure 2A). Moreover, the sensitization level compositions of peanuts were significant difference between healthy males and females (*χ*^2^ test, *χ*^2^ = 7.150, *P* = 0.028; Figure 2A).

Notably, compared with healthy cases, the proportion of high-level (≥ Level 3) sensitization in allergic cases was relatively higher, and the level composition of sensitization caused by milk in girls with allergies was significantly different from that in healthy cases (*χ*^2^ test, *χ*^2^ = 16.687, *P* = 0.005; Figure 2A). The level composition of sensitization caused by milk in boys with allergies was significantly different from that in healthy cases (*χ*^2^ test, *χ*^2^ = 9.981, *P* = 0.041; Figure 2A). Additionally, the level composition of peanut and beef sensitization in boys with RIDs was significantly different from that in healthy cases (*χ*^2^ test, *P* < 0.05; Figure 2A).

### Distribution differences of allergen sources between sexes

We subsequently analyzed the differences of specific allergen sensitization between sexes in the 1,368 children who were ultimately diagnosed with allergic diseases. Sensitization rates for tree combinations, *Ambrosia artemisiifolia*, cat fur, and peanuts exhibited significant difference between sexes (*χ*^2^ test, *P* < 0.05; Table 1). The sensitization rates of these allergy sources in boys were all significantly higher than those of girls (*χ*^2^ test, *P* < 0.05; Table 1).

**Table 1:** Sensitization differences of allergen sources between sexes.

Allergen source	Male (*n* = 747)	Female (*n* = 621)	*χ*2	*P*
Tree combinations	145 (19.41%, 16.63% - 22.43%)	80 (12.88%, 10.35% - 15.78%)	10.047	0.002
*Ambrosia artemisiifolia*	80 (10.71%, 8.58% - 13.15%)	42 (6.76%, 4.92% - 9.03%)	6.024	0.014
*Artemisia argyi*	83 (11.11%, 8.95% - 13.59%)	52 (8.37%, 6.32% - 10.84%)	2.558	0.110
Combinations of indoor *Dermatophagoides*	12 (1.61%, 0.83% - 2.79%)	7 (1.13%, 0.45% - 2.31%)	0.273	0.602
House dust	26 (3.48%, 2.29% - 5.067%)	13 (2.09%, 1.12% - 3.55%)	1.882	0.170
Cat fur	102 (13.65%, 11.27% - 16.33%)	60 (9.66%, 7.45% - 12.26%)	4.803	0.028
Dog epithelium	31 (4.15%, 2.84% - 5.84%)	23 (3.70%, 2.36% - 5.51%)	0.080	0.778
*Periplaneta americana*	23 (3.08%, 1.96% - 4.58%)	17 (2.74%, 1.60% - 4.35%)	0.045	0.832
Mold combinations	27 (3.61%, 2.40% - 5.22%)	29 (4.67%, 3.15% - 6.64%)	0.712	0.399
*Humulus scandens*	38 (5.09%, 3.62% - 6.92%)	20 (3.22%, 1.98% - 4.93%)	2.468	0.116
Egg whites	86 (11.51%, 9.31% - 14.02%)	58 (9.34%, 7.17% - 11.91%)	1.477	0.224
Milk	46 (6.16%, 4.54% - 8.13%)	30 (4.83%, 3.28% - 6.82%)	0.899	0.343
Peanuts	49 (6.56%, 4.89% - 18.58%)	24 (3.86%, 2.49% - 5.70%)	4.356	0.037
Soybeans	27 (3.61%, 2.40% - 5.22%)	25 (4.03%, 2.62% - 5.89%)	0.065	0.799
Beef	22 (2.95%, 1.85% - 4.43%)	18 (2.90%, 1.73% - 4.54%)	6.238 × 10^−29^	1
Seafood combinations	5 (0.67%, 0.22% - 1.56%)	4 (0.64%, 0.18% - 1.64%)	1.148 × 10^−31^	1
Shrimps	10 (1.34%, 0.64% - 2.45%)	6 (0.97%, 0.36% - 2.09%)	0.149	0.700
Crabs	10 (1.34%, 0.64% - 2.45%)	6 (0.97%, 0.36% - 2.09%)	0.149	0.700
Mutton	33 (4.42%, 3.06% - 6.15%)	25 (4.03%, 2.62% - 5.89%)	0.050	0.823

The data were shown as *n* (Positive rate, 95% CI).

### Distribution differences of allergen sources among ages

**Figure 3:**
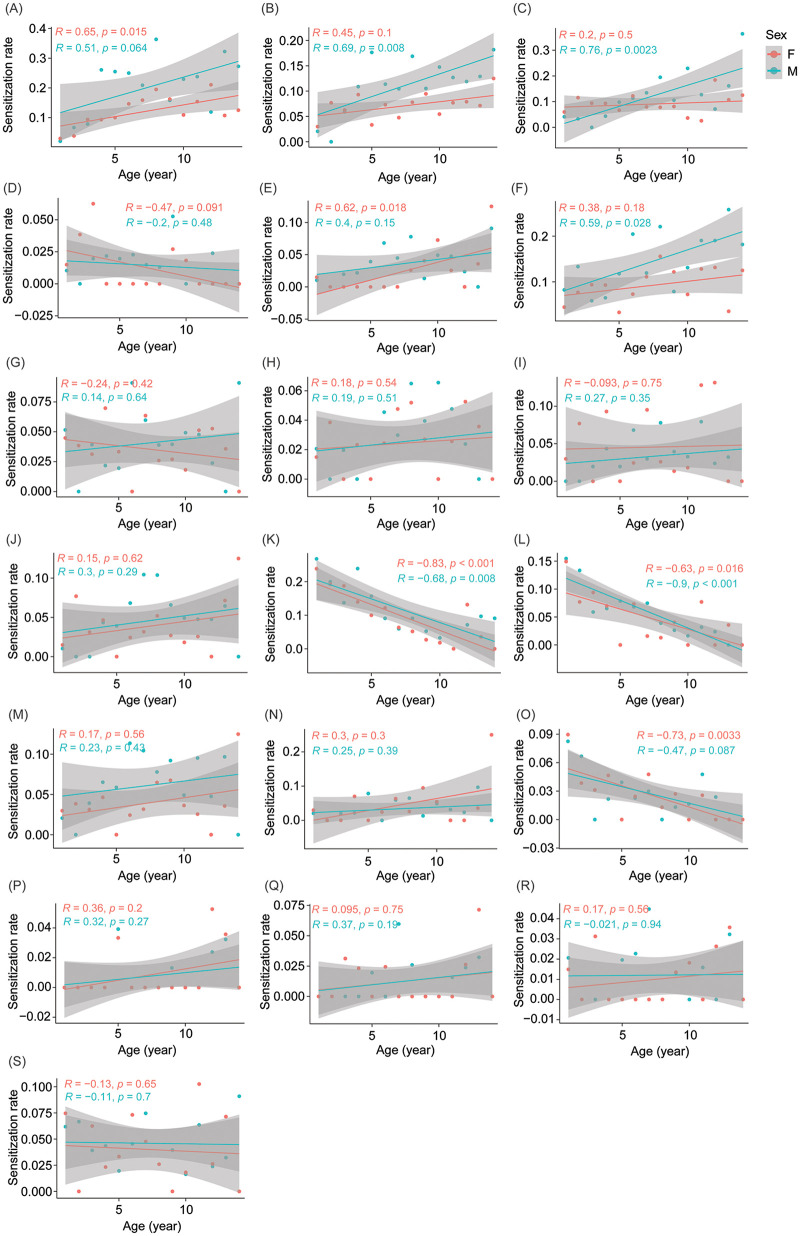
Correlation between age and sensitization rate of specific allergen in children. **(A)** Tree combinations; **(B)** *Ambrosia artemisiifolia*; **(C)** *Artemisia argyi*; **(D)** Combinations of indoor *Dermatophagoides*; **(E)** House dust; **(F)** Cat fur; **(G)** Dog epithelium; **(H)** *Periplaneta americana*; **(I)** Mold combinations; **(J)** *Humulus scandens*; **(K)** Egg whites; **(L)** Milk; **(M)** Peanuts; **(N)** Soybeans; **(O)** Beef; **(P)** Seafood combinations; **(Q)** Shrimps; **(R)** Crabs; **(S)** Mutton.

We then analyzed the correlation between allergen sensitization and age in the children who were ultimately diagnosed with allergic diseases. Allergy sensitization rates of tree combinations, *Ambrosia artemisiifolia*, *Artemisia argyi*, house dust, cat fur, mold combinations, *Humulus scandens*, egg whites, milk, and beef exhibited significant differences among age groups (*χ*^2^ test, *P* < 0.05; Table 2). Interestingly, tree combinations, *Ambrosia artemisiifolia*, *Artemisia argyi*, house dust, cat fur, mold combinations, and *Humulus scandens* exhibited increasing trends in sensitization rate with age, whereas egg whites, milk, and beef exhibited decreasing trends in sensitization rate with age (Table 3). Further correlation analysis revealed that the sensitization rates of each allergy sources changed with age were roughly consistent between boys and girls (Figure 3). Especially, the allergy sensitization rates of tree combinations (*R* = 0.65, *P* = 0.015; Figure 3A), and house dust (*R* = 0.62, *P* = 0.018; Figure 3E) increased significantly with age in girls, whereas the allergy sensitization rates of *Ambrosia artemisiifolia* (*R* = 0.69, *P* = 0.008; Figure 3B), *Artemisia argyi* (*R* = 0.76, *P* = 0.002; Figure 3C), cat fur (*R* = 0.59, *P* = 0.028; Figure 3F) increased significantly with age in boys. Moreover, the allergy sensitization rates of egg whites (*R* = −0.83, *P* < 0.001; Figure 3 K), milk (*R* = −0.63, *P* = 0.016; Figure 3L), and beef (*R* = −0.73, *P* = 0.003; Figure 3O) decreased significantly with age in girls, and the allergy sensitization rates of egg whites (*R* = −0.68, *P* = 0.008; Figure 3 K), and milk (*R* = −0.90, *P* < 0.001; Figure 3L) decreased significantly with age in boys. These results suggested that the sources of inhalant allergens were more likely to increase with age, whereas the sources of food allergens were more likely to decrease.

**Table 2:** Distribution of allergen sources in different age groups.

Allergen sources	Toddler (*n* = 303)	Preschool (*n* = 255)	School-age (*n* = 810)	*χ* ^2^	*P*
Tree combinations	14 (4.62%, 2.55% - 7.63%)	49 (19.22%, 14.57% - 24.60%)	162 (20.00%, 17.30% - 22.92%)	39.702	<0.001
*Ambrosia artemisiifolia*	11 (3.63%, 1.83% - 6.40%)	27 (10.59%, 7.09% - 15.03%)	84 (10.37%, 8.36% - 12.68%)	13.409	0.001
*Artemisia argyi*	15 (4.95%, 2.80% - 8.03%)	23 (9.02%, 5.80% - 13.23%)	97 (11.98%, 9.82% - 14.41%)	12.488	0.002
Combinations of indoor *Dermatophagoides*	6 (1.98%, 0.73% - 4.26%)	3 (1.18%, 0.24% - 3.40%)	10 (1.23%, 0.59% - 2.26%)	0.998	0.607
House dust	3 (0.99%, 0.20% - 2.87%)	6 (2.35%, 0.87% - 5.05%)	30 (3.70%, 2.51% - 5.25%)	6.143	0.046
Cat fur	23 (7.59%, 4.87% - 11.17%)	26 (10.20%, 6.77% - 14.58%)	113 (13.95%, 11.64% - 16.53%)	9.357	0.009
Dog epithelium	12 (3.96%, 2.06% - 6.82%)	10 (3.92%, 1.90% - 7.09%)	32 (3.95%, 2.72% - 5.53%)	0.001	0.999
*Periplaneta americana*	5 (1.65%, 0.54% - 3.81%)	4 (1.57%, 0.43% - 3.97%)	31 (3.83%, 2.61% - 5.39%)	5.710	0.058
Mold combinations	5 (1.65%, 0.54% - 3.81%)	11 (4.31%, 2.17% - 7.59%)	40 (4.94%, 3.55% - 6.66%)	6.111	0.047
*Humulus scandens*	5 (1.65%, 0.54% - 3.81%)	10 (3.92%, 1.90% - 7.09%)	43 (5.31%, 3.87% - 7.08%)	7.348	0.025
Egg whites	66 (21.78%, 17.27% - 26.86%)	37 (14.51%, 10.43% - 19.44%)	41 (5.06%, 3.66% - 6.80%)	70.738	<0.001
Milk	37 (12.21%, 8.75% - 16.44%)	16 (6.27%, 3.63% - 9.99%)	23 (2.84%, 1.81% - 4.23%)	37.221	<0.001
Peanuts	8 (2.64%, 1.15% - 5.14%)	14 (5.49%, 3.03% - 9.04%)	51 (6.30%, 4.72% - 8.20%)	5.850	0.054
Soybeans	5 (1.65%, 0.54% - 3.81%)	9 (3.53%, 1.63% - 6.59%)	38 (4.69%, 3.34% - 6.38%)	5.641	0.060
Beef	18 (5.94%, 3.56% - 9.23%)	7 (2.75%, 1.11% - 5.57%)	15 (1.85%, 1.04% - 3.04%)	13.023	0.001
Seafood combinations	0 (0%, 0% - 1.21%)	3 (1.18%, 0.24% - 3.40%)	6 (0.74%, 0.27% - 1.61%)	3.141	0.208
Shrimps	1 (0.33%, 0.01% - 1.83%)	3 (1.18%, 0.24% - 3.40%)	12 (1.48%, 0.77% - 2.57%)	2.529	0.282
Crabs	4 (1.32%, 0.36% - 3.35%)	2 (0.78%, 0.10% - 2.80%)	10 (1.23%, 0.59% - 2.26%)	0.416	0.812
Mutton	17 (5.61%, 3.30% - 8.83%)	10 (3.92%, 1.90% - 7.09%)	31 (3.83%, 2.61% - 5.39%)	1.806	0.405

The data were shown as *n* (positive rate, 95% CI). Toddler, children with 0 to ≤3 years of ages; preschool, children with 4 to ≤6 years of ages; School-age, children with 7 to ≤14 years of ages.

**Table 3:** Distributions of allergen sources in seasons.

Allergen source	Spring (*n* = 437)	Summer (*n* = 285)	Autumn (*n* = 387)	Winter (*n* = 259)	*χ*2	*P*
Tree combinations	105 (24.03%, 20.09% - 28.32%)	52 (18.25%, 13.94% - 23.23%)	44 (11.37%, 8.38% - 14.96%)	24 (9.27%, 6.03% - 13.47%)	35.922	<0.001
*Ambrosia artemisiifolia*	35 (8.01%, 5.64% - 10.96%)	34 (11.93%, 8.40% - 16.27%)	38 (9.82%, 7.04% - 13.23%)	15 (5.79%, 3.28% - 9.37%)	7.131	0.068
*Artemisia argyi*	52 (11.90%, 9.02% - 15.31%)	28 (9.82%, 6.63% - 13.89%)	37 (9.56%, 6.82% - 12.94%)	18 (6.95%, 4.17% - 10.76%)	4.549	0.208
Combinations of indoor *Dermatophagoides*	6 (1.37%, 0.51% - 2.96%)	7 (2.46%, 0.99% - 4.99%)	2 (0.52%, 0.06% - 1.85%)	4 (1.54%, 0.42% - 3.91%)	4.566	0.207
House dust	6 (1.37%, 0.51% - 2.96%)	16 (5.61%, 3.24% - 8.96%)	10 (2.58%, 1.25% - 4.70%)	7 (2.70%, 1.09% - 5.49%)	11.423	0.010
Cat fur	41 (9.38%, 6.82% - 12.51%)	44 (15.44%, 11.45% - 20.17%)	50 (12.92%, 9.74% - 16.68%)	27 (10.42%, 6.98% - 14.80%)	6.993	0.072
Dog epithelium	16 (3.66%, 2.11% - 5.88%)	17 (5.96%, 3.51% - 9.38%)	14 (3.62%, 1.99% - 6.00%)	7 (2.70%, 1.09% - 5.49%)	4.323	0.229
*Periplaneta americana*	9 (2.06%, 0.95% - 93.87%)	10 (3.51%, 1.70% - 6.36%)	16 (4.13%, 2.38% - 6.63%)	5 (1.93%, 0.63% - 4.45%)	4.392	0.222
Mold combinations	16 (3.66%, 2.11% - 5.88%)	15 (5.26%, 2.98% - 8.53%)	14 (3.62%, 1.99% - 6.00%)	11 (4.25%, 2.14% - 7.47%)	1.440	0.696
*Humulus scandens*	20 (4.58%, 2.82% - 6.98%)	13 (4.56%, 2.45% - 7.67%)	15 (3.88%, 2.19% - 6.31%)	10 (3.86%, 1.87% - 6.99%)	0.412	0.938
Egg white	46 (10.53%, 7.81% - 13.79%)	39 (13.68%, 9.92% - 18.23%)	36 (9.30%, 6.60% - 12.65%)	23 (8.88%, 5.71% - 13.03%)	4.378	0.223
Milk	27 (6.18%, 4.11% - 8.86%)	19 (6.67%, 4.06% - 10.22%)	19 (4.91%, 2.98% - 7.56%)	11 (4.25%, 2.14% - 7.47%)	2.147	0.543
Peanuts	25 (5.72%, 3.74% - 8.33%)	21 (7.37%, 4.62% - 11.04%)	21 (5.43%, 3.39% - 8.18%)	6 (2.32%, 0.85% - 4.97%)	7.139	0.068
Soybeans	17 (3.89%, 2.28% - 6.16%)	12 (4.21%, 2.19% - 7.24%)	18 (4.65%, 2.78% - 7.25%)	5 (1.93%, 0.63% - 4.45%)	3.383	0.336
Beef	13 (2.97%, 1.59% - 5.03%)	13 (4.56%, 2.45% - 7.67%)	11 (2.84%, 1.43% - 5.03%)	3 (1.16%, 0.24% - 3.35%)	5.550	0.136
Seafood combinations	3 (0.69%, 0.14% - 1.99%)	2 (0.70%, 0.09% - 2.51%)	3 (0.78%, 0.16% - 2.25%)	1 (0.39%, 0.01% - 2.13%)	0.388	0.943
Shrimps	6 (1.37%, 0.51% - 2.96%)	4 (1.40%, 0.38% - 3.55%)	3 (0.78%, 0.16% - 2.25%)	3 (1.16%, 0.24% - 3.35%)	0.812	0.847
Crabs	5 (1.14%, 0.37% - 2.65%)	2 (0.70%, 0.09% - 2.51%)	5 (1.29%, 0.42% - 2.99%)	4 (1.54%, 0.42% - 3.91%)	0.907	0.824
Mutton	12 (2.75%, 1.43% - 4.75%)	5 (1.75%, 0.57% - 4.05%)	21 (5.43%, 3.39% - 8.18%)	20 (7.72%, 4.78% - 11.67%)	15.815	0.001

The data were shown as *n* (Positive rate, 95% CI).

### Distribution differences of allergen sources among seasons

Finally, we analyzed the distribution differences of allergy sensitization among seasons in children who were ultimately diagnosed with allergic diseases. In the 19 tested allergens, the sensitization rates of those from tree combinations (*χ*^2^ test, *χ*^2^ = 35.922, *P* < 0.001), house dust (*χ*^2^ test, *χ*^2^ = 11.423, *P* = 0.010), and mutton (*χ*^2^ test, *χ*^2^ = 15.815, *P* = 0.001) exhibited significant seasonal differences (Table 3). The highest sensitization for tree combinations was in the spring (24.03%, 95% CI 20.09% - 28.32%), whereas the lowest was in the winter (9.27%, 95% CI 6.03% - 13.47%). However, the highest sensitization for house dust was in the summer (5.61%, 95% CI 3.24% - 8.96%), and the lowest was in the spring (1.37%, 95% CI 0.51% - 2.96%). For mutton, the lowest sensitization was in the summer (1.75%, 95% CI 0.57% - 4.05%), and the highest was in the winter (7.72%, 95% CI 4.78% - 11.67%).

## Discussion

The prevalence of allergic diseases in children has increased recently, posing a threat to their normal life and learning (13). These diseases are chronic and have complex causes, making clinical treatment difficult and increasing the likelihood of recurrence. Allergic diseases are typically mediated by IgE, and individuals with allergies are more likely to produce IgE in response to environmental allergens. The distribution and levels of these allergens are influenced by environmental factors and lifestyle choices (7). These levels also change dynamically with the development of society (14–16). However, there is a lack of research on the distribution of allergens and the factors that influence them in the population living in Kashi Prefecture. Therefore, we determine the rate of sIgE-sensitization inhalant allergens in children under 14 years old with suspected allergic diseases in Kashi Prefecture. The results of this study provide a basis for the diagnosis, treatment, and prevention of these diseases.

The sources of allergens and their sensitization rates vary across different geographic regions. For instance, in southwest Shanghai, China, the main allergen sources were *Dermatophagoides farinae* and *Dermatophagoides pteronyssinus* (17), whereas in Suzhou, China, the dominant allergens were a combination of *Dermatophagoides*, house dust, and molds (18). In Yinchuan, China, the highest sensitization rate was for mold combination (19), followed by cat and dog fur, *Ambrosia artemisiifolia* and *Artemisia argyi*, *Dermatophagoides farinae* and *Dermatophagoides pteronyssinus*, and *Humulus scandens*. In Beijing, China, the top four inhalant allergen sources were *Candida*/*Penicillium notatum*/*Cladosporium*/*Alternaria*/*Aspergillus niger*, cat fur, dog epithelium, and *Dermatophagoides pteronyssinus*, whereas the main food allergen sources were milk, egg whites, egg yolk, and wheat/buckwheat (20). Our study in Kashi Prefecture showed that the main inhalant allergens were tree combinations (330, 15.54%), cat fur (243, 11.44%), and *Artemisia argyi* (195, 9.18%), whereas the main food allergens were egg whites (229, 10.78%), milk (76, 5.56%), and peanuts (73, 5.34%) (Figure 1). These regional differences may be attributed to climatic conditions, lifestyles, and eating habits. For instance, the humid areas in the south are more conducive to the breeding of dust mites and molds, whereas the unique climate and vegetation distribution in Kashi Prefecture make plant allergens such as tree pollen and mugwort more prevalent. These variations suggest the need to consider local epidemiological characteristics and develop targeted prevention strategies when testing and addressing allergen sources.

There are significant differences in the sensitization rate of allergens among children of different ages. The positive rate of food allergens was higher in children aged 0–3 years old, specifically egg white, milk, and beef. However, the sensitization rate of inhalant allergens was lower (Table 2), which was consistent with studies conducted in other regions of China (21–23). This may be due to the underdeveloped protective function of the gastrointestinal mucosa in infants, making it easier for macromolecular proteins in food to pass through gastrointestinal mucosa and cause allergic reactions. Additionally, younger children aged 0–3 years old tend to have less outdoor activities and limited exposure to inhalant allergens. In contrast, the positive rate of inhalant allergens significantly increased in children over 6 years old, possibly due to their increased exposure to these allergens as they age. Furthermore, the immune system of older children gradually matures, making them more responsive to inhalant allergens. Therefore, targeted allergen detection and preventive measures should be taken for children of different ages. Younger children should focus on managing food allergies, whereas older children should prioritize interventions in their living environment to reduce exposure to inhalant allergens.

Generally, boys have a higher positive rate of allergens and a higher prevalence of allergic diseases compared to girls (24, 25). For instance, in Shanghai, China, the positive rates of *Dermatophagoides farinae* and *Dermatophagoides pteronyssinus* were significantly higher in boys than in girls (17). A study in Nanjing, China also found that the overall allergen positive rate in boys was significantly higher than in girls, with a more noticeable difference in the positive rates of *Dermatophagoides* and *Periplaneta americana* (23). Our study also showed that the sensitization rates of allergens from tree combinations, *Ambrosia artemisiifolia*, cat fur, and peanuts were significantly higher in boys than in girls (*P* < 0.05; Table 1). This difference in sex may be attributed to the hormone levels in boys, such as androgen enhancing Th2 immune response, and the possibility that boys' immune systems may be more sensitive to allergens (24, 26).

The prevalence of allergens shows a clear seasonal pattern. In Chengdu, China, the positive rate of *Dermatophagoides pteronyssinus* was significantly higher in summer compared to other seasons (27). This could be attributed to the hot and humid weather during this time. Similarly, a study in Fuzhou, China found that the peak of positive rate of inhalant allergens occurred from April to October, which coincides with the breeding season of *Dermatophagoides* and pollen (28). These findings suggest that environmental factors play a significant role in triggering allergies and highlight the need for increased environmental intervention and health education during peak allergy seasons. It is worth noting that the sources of inhalant allergens were more likely to trigger allergies in the spring, whereas the sources of food allergens were more likely to trigger allergies in the autumn. This highlights the importance of targeted interventions and education during specific seasons. Additionally, prevalent compositions of allergen sources vary across different regions. For instance, wet areas in South China are more prone to *Dermatophagoides* and mold allergies (17, 18, 29), whereas dry areas in North China have a higher prevalence of pollen and pet hair allergies (19). Furthermore, coastal and southern regions of China have a higher prevalence of egg white and milk allergies, whereas shrimp and crab allergies are more common in Southwest China (28, 30). Our study in Kashi Prefecture, a dry area, found that plant allergens (such as mugwort) and pet hair allergies were more prevalent. Understanding these regional differences is crucial in implementing effective allergen prevention and intervention measures.

Notably, allergy is clinically identified through the symptoms that occur upon exposure to allergens (31). A person is considered sensitized if sIgE antibodies are found in their serum or plasma, or if they have a positive skin test, even if they have not shown any clinical reactions (31). Sensitization is a necessary condition and a risk factor for developing allergies, but it does not equate to an allergy diagnosis (31). For an accurate allergy diagnosis, sIgE test results must be evaluated alongside the patient's medical history and clinical symptoms (31). Although sIgE can be present without allergy symptoms, evidence indicates that sIgE levels between 0.1 and 0.35 kUA/L might be clinically significant for some individuals, particularly children, though further investigation is needed for various allergies (31–42). In this study, our results showed that even without allergic symptoms, children considered healthy also showed sensitization for common allergens, especially the sensitization for tree combinations. However, compared with children with allergic symptoms, the level of sensitization in healthy children was relatively low (Figure 2).

The results of this study suggest that specific IgE detection can effectively identify allergens in children, providing a crucial foundation for the diagnosis of allergic diseases. By identifying specific allergens, targeted intervention measures can be implemented, such as avoiding contact with allergens and specific immunotherapy, to reduce the incidence of allergic diseases. Additionally, medical staff should conduct targeted allergen detection and prevention for children based on the local allergen epidemiological characteristics, to improve the quality of life for children. However, there are some limitations to this study. Firstly, the sample size is limited to Kashi Prefecture and may not fully reflect the distribution of allergens in other regions. Secondly, the cross-reactivity of allergens was not thoroughly analyzed, which may affect the accurate evaluation of allergen sensitization intensity. Future research should consider expanding the sample range and incorporating multicenter data to further investigate the distribution of allergens and their relationship with clinical symptoms.

## Conclusion

Sensitization to common allergens was very common among children aged 0–14 years in Kashi Prefecture, but only a few children exhibited allergic symptoms. Children exhibited notable variations in the sensitization of sIgE allergens based on age, sex, and season. To prevent allergic diseases in these children, it is essential to develop allergen source prevention strategies tailored to different demographics and seasons. Nonetheless, further investigation into the mechanisms behind these differences is necessary to more effectively create a prevention fremework for allergic diseases in children in the Kashi Prefecture.

## Data Availability

The original contributions presented in the study are included in the article/Supplementary Material, further inquiries can be directed to the corresponding author.
