# Supplementary material for: Sensitization to 19 allergen sources in 2,124 children in Kashi Prefecture, China: a single-center cross-sectional retrospective study
Source: Front Allergy. 2026 Apr 29;7:1795685. doi: 10.3389/falgy.2026.1795685 (PMC13168159; doi:10.3389/falgy.2026.1795685)
Supplement: Supplementary file 2 [file Table2.docx]

Table S2. Number of children included in each age group. Toddler group, 0 to ≤3 years old; preschool group, 4 to ≤6 years old; school-age group, 7 to ≤14 years old. RIDs, respiratory infectious diseases.

| Age group | Allergic diseases | | Health | | RIDs | | All cases | | Total |
| --- | --- | --- | --- | --- | --- | --- | --- | --- | --- |
|  | Male | Female | Male | Female | Male | Female | Male | Female |  |
| Toddler | 178 | 125 | 44 | 37 | 92 | 59 | 314 | 221 | 535 |
| Preschool | 141 | 114 | 17 | 11 | 91 | 69 | 249 | 194 | 443 |
| School age | 428 | 382 | 31 | 21 | 154 | 123 | 613 | 526 | 1139 |
| *χ*^2^ | 3.163 | | 0.539 | | 1.153 | | 3.598 | |  |
| *P* | 0.206 | | 0.764 | | 0.562 | | 0.166 | |  |
