# Supplementary material for: Sensitization to 19 allergen sources in 2,124 children in Kashi Prefecture, China: a single-center cross-sectional retrospective study
Source: Front Allergy. 2026 Apr 29;7:1795685. doi: 10.3389/falgy.2026.1795685 (PMC13168159; doi:10.3389/falgy.2026.1795685)
Supplement: Supplementary file 3 [file Table3.docx]

Table S3. Number of children included in each season. RIDs, respiratory infectious diseases; VSDs, viral skin diseases.

| Season | Allergic diseases | | Health | | RIDs | | VSDs | | All cases | | Total |
| --- | --- | --- | --- | --- | --- | --- | --- | --- | --- | --- | --- |
|  | Male | Female | Male | Female | Male | Female | Male | Female | Male | Female |  |
| Spring | 243 | 194 | 26 | 17 | 105 | 78 | 1 | 1 | 375 | 290 | 665 |
| Summer | 155 | 130 | 24 | 20 | 98 | 62 | 4 | 1 | 281 | 213 | 494 |
| Autumn | 207 | 180 | 21 | 16 | 72 | 64 | 0 | 0 | 300 | 260 | 560 |
| Winter | 142 | 117 | 21 | 16 | 62 | 47 | 0 | 0 | 225 | 180 | 405 |
| *χ*^2^ | 0.382 | | 0.320 | | 2.085 | | 1.553×10^-31^ | | 1.432 | |  |
| *P* | 0.944 | | 0.956 | | 0.555 | | 1.000 | | 0.698 | |  |
